# Supplementary material for: Performance evaluation of Biofire Film Array Respiratory Panel 2.1 for SARS-CoV-2 detection in a pediatric hospital setting
Source: PLoS One. 2023 Oct 5;18(10):e0292314. doi: 10.1371/journal.pone.0292314 (PMC10553804; doi:10.1371/journal.pone.0292314)
Supplement: Attachment — Submitted filename: Response to Reviewers.pdf [file pone.0292314.s002.pdf]

**Response to Editor and Reviewers**

We appreciate the constructive feedback and guidance from the editor, as well as the comments from the reviewers. We are confident that these constructive inputs will significantly enhance the quality of our manuscript.

1. When submitting your revision, we need you to address these additional requirements. Please ensure that your manuscript meets PLOS ONE's style requirements, including those for file naming. The PLOS ONE style templates can be found at https://journals.plos.org/plosone/s/file?id=wjVg/PLOSOne_formatting_sample_main_body.pdf and https://journals.plos.org/plosone/s/file?id=ba62/PLOSOne_formatting_sample_title_authors_affiliations.pdf

**Answer**

We have carefully reviewed the additional requirements and have made the necessary revisions to ensure that our manuscript complies with PLOS ONE's style requirements, including file naming. We have used the provided PLOS ONE style templates to guide us in this process.

2. Thank you for stating in your Funding Statement:
This study was financially supported by Consejo Nacional de Ciencia y Tecnología, CONACYT, Paraguay (grant number PINV 20-287), https://www.conacyt.gov.py/. The funder had no role in study design, data collection and analysis, decision to publish, or preparation of the manuscript.
Please provide an amended statement that declares *all* the funding or sources of support (whether external or internal to your organization) received during this study, as detailed online in our guide for authors at http://journals.plos.org/plosone/s/submit-now. Please also include the statement “There was no additional external funding received for this study.” in your updated Funding Statement.
Please include your amended Funding Statement within your cover letter. We will change the online submission form on your behalf.

**Answer**

We provided an amended statement that declares all the funding or sources of support received during this study. We also included the statement 'There was no additional external funding received for this study' in our updated Funding Statement within our cover letter.

3. In your Data Availability statement, you have not specified where the minimal data set underlying the results described in your manuscript can be found. PLOS defines a study's minimal data set as the underlying data used to reach the conclusions drawn in the manuscript and any additional data required to replicate the reported study findings in their entirety. All PLOS journals require that the minimal data set be made fully available. For more information about our data policy, please see http://journals.plos.org/plosone/s/data-availability.

Upon re-submitting your revised manuscript, please upload your study's minimal underlying data set as either Supporting Information files or to a stable, public repository and include the relevant URLs, DOIs, or accession numbers within your revised cover letter. For a list of acceptable repositories, please see http://journals.plos.org/ plosone/s/data-availability#loc-recommended-repositories. Any potentially identifying patient information must be fully anonymized.
Important: If there are ethical or legal restrictions to sharing your data publicly, please explain these restrictions in detail. Please see our guidelines for more information on what we consider unacceptable restrictions to publicly sharing data: http://journals.plos.org/plosone/s/data-availability#loc-unacceptable-data-access-restrictions. Note that it is not acceptable for the authors to be the sole named individuals responsible for ensuring data access.
We will update your Data Availability statement to reflect the information you provide in your cover letter.

**Answer**

In the Data Availability Statement, we have mentioned that "All relevant data are within the manuscript and its Supporting Information files". Additionally, we have uploaded as a supporting file the Data Base including anonymized participants' demographic, clinical, and laboratory characteristics, where the minimal data set underlying the results described in this manuscript can be found.

4. Please review your reference list to ensure that it is complete and correct. If you have cited papers that have been retracted, please include the rationale for doing so in the manuscript text, or remove these references and replace them with relevant current references. Any changes to the reference list should be mentioned in the rebuttal letter that accompanies your revised manuscript. If you need to cite a retracted article, indicate the article's retracted status in the References list and also include a citation and full reference for the retraction notice.

**Answer**

References were reviewed and formatted according to the Plos One journal requirements.

**Reviewer #1:**
This is a useful study demonstrating the clinical utility of BioFire Respiratory panel for the detection of SARS-CoV-2. The study is well-performed and the paper is well written.

Thank you for your thoughtful review and positive feedback regarding our study. We appreciate your valuable comments.

1. The authors should cite more extensively to introduce the pathogenesis and clinical significance based on the following references.
Liu BM, Martins TB, Peterson LK, Hill HR. Clinical significance of measuring serum cytokine levels as inflammatory biomarkers in adult and pediatric COVID-19 cases: A review. Cytokine. 2021 Jun;142:155478. doi: 10.1016/j.cyto.2021.155478. Epub 2021 Feb 23. PMID: 33667962; PMCID: PMC7901304.

Hu B, Huang S, Yin L. 2020. The cytokine storm and COVID-19. J Med Virol. DOI: 10.1002/jmv.26232.
Liu B, Hill HR. Role of Host Immune and Inflammatory Responses in COVID-19 Cases with Underlying Primary Immunodeficiency: A Review. J Interferon Cytokine Res. 2020 Dec;40(12):549-554. doi: 10.1089/jir.2020.0210. PMID: 33337932; PMCID: PMC7757688.

**Answer**

We concur with the significance of introducing the role of cytokines in the pathogenesis of COVID-19 within the introduction section. We have emphasized the great relevance of this matter and integrated the suggested references (lines 66-71).

2. Please clarify inclusion and exclusion criteria

**Answer**

The inclusion and exclusion criteria were clarified in the text (lines 142-144).

3. Is the statistical analysis two-tailed or one-tailed?

**Answer**

All statistical analyses were conducted using a two-tailed approach. This clarification has been included in the text for clarity (lines 246-247).

4. In the Introduction or Discussion, please discuss the following point based on the following reference: though the BioFire Respiratory panel is a large multiplex PCR panel, it still lacks some important respiratory pathogen to be covered, e.g., Pneumocystis jirovecii, which can be detected otherwise by some other novel molecular assays.
Liu B, Totten M, Nematollahi S, Datta K, Memon W, Marimuthu S, Wolf LA, Carroll KC, Zhang SX. Development and Evaluation of a Fully Automated Molecular Assay Targeting the Mitochondrial Small Subunit rRNA Gene for the Detection of Pneumocystis jirovecii in Bronchoalveolar Lavage Fluid Specimens. J Mol Diagn. 2020 Dec;22(12):1482- 1493. doi: 10.1016/j.jmoldx.2020.10.003. Epub 2020 Oct 15. Erratum in: J Mol Diagn. 2021 Apr;23(4):506. PMID: 33069878.
5. In the Introduction or Discussion, please discuss that BioFire Respiratory panel provides some level of commutablity and standardization in different clinical labs as the same platform and panel is being used. According to the following reference, different reverse transcriptase with varied efficiency may cause unreliable results. BioFire Respiratory panel solves this problem as a popular and widely used commercial molecular test. Please discuss this based on this reference.
Liu B, Forman M, Valsamakis A. Optimization and evaluation of a novel real-time RT-PCR test for detection of parechovirus in cerebrospinal fluid. J Virol Methods. 2019 Oct;272:113690. doi: 10.1016/j.jviromet.2019.113690. Epub 2019 Jul 5. PMID: 31283959.

**Answer**

In response to these two comments (4 and 5), we respectfully disagree with the reviewer regarding the relevance of incorporating the suggested references into our study discussion. The first reference pertains to the development of an assay for the detection of

Pneumocystis jirovecii in Bronchoalveolar samples, a topic not within the scope of our study, which exclusively evaluates the BioFire Respiratory panel for SARS-CoV-2 detection.
Similarly, the second reference concerns parechovirus detection, which is unrelated to the subject of our study.
Consequently, we believe these references do not align with the focus of our research and, therefore, cannot be reasonably discussed in our study.

6. Error: Line 57: "Wuhan province" should be "Wuhan city"

**Answer**

The correction has been implemented.

**Additional clarification**

We would like to clarify that there was a typographical error in our previous statement regarding the BioFire FA-RP2.1 specificity. The accurate specificity for this test is 94%, not 75%. As a result, we have made the necessary revisions to the discussion section (lines 327-330).
